# Legal

**Published:** 1870-02

**Authors:** 


### Editorial.

### LEGAL.

We give below, in full, a Bill now before the Legislature of Iowa for the suppression of Dental quackery, and the encouragement of scientific attainments in the profession of Dentistry.

The Bill we regard as a very good one, and should be enacted. It is so framed as to cover all the material points. There are some features in this Bill that will meet the objections made to the laws in other states. For instance, in Section 6: "Any member of the Board of Examiners, on satisfactory examination of applicant, shall grant permission to practice until a regular meeting of the Board."

This is a very good regulation; it is an enabling clause that every such law should have.

It will be seen, too, that none are exempt from the requirements of the law, and that it becomes operative upon all at once. Jan. 1, 1871. It might be better that a little longer time should be given, at least, to a certain class of practitioners.

Similar bills are now pending before the Legislatures of several States, and we doubt not that during the present year there will be Dental laws in force in some of these, at least; and if they are so framed that each shall receive as definite benefit as seems accruing in Ohio, then will it be a matter of great congratulation for the whole profession indeed.

The wave of progress rolls on with resistless power, notwithstanding some few, with mops, try to keep it back.

### A BILL

*For the Suppression of Dental Quackery and the Encouragement of Scientific Attainments in the Profession of Dentistry.*

SECTION 1. *Be it enacted by the General Assembly of the State of Iowa,* That it shall be unlawful for any person to practice Dentistry for compensation, unless such person has received a diploma from the faculty of a Dental College, duly incorporated under the laws of this or any other State of these United States, or foreign country, and duly recognized by the American Dental Association, or has a certificate of qualification from a board of examiners hereinafter provided, or has a certificate of membership of the Iowa State Dental Society.

SEC. 2. The Governor shall appoint persons recommended by the Iowa State Dental Society, who shall constitute said board of examiners, whose term of office shall continue for three years, and until their successors are appointed.

SEC. 3. Said board shall consist of six persons, who must be graduates of some regular dental or medical college, or possess the evidence of qualification contemplated in this act, and be in regular daily practice of dental surgery.

SEC. 4. The following shall be the division of said board, namely:

One on Anatomy and Oral Surgery.
One on Dental Physiology and Histology.
One on Dental Pathology and Therapeutics.
One on Dental Chemistry.
One on Operative Dentistry and Hygiene.
One on Mechanical Dentistry and Metallurgy.

SEC. 5. Said board shall hold annual sessions for the purpose of examining applicants, at such times and places as the Iowa State Dental Society may appoint, and shall give public notice of such meeting for at least sixty days.

SEC. 6. Any member of the board of examiners, on satisfactory examination of applicant, shall grant a permission to practice until the regular meeting of the board.

SEC. 7. Each applicant shall, on receiving a certificate, pay into the treasury of the Iowa State Dental Society the sum of ten dollars, which shall be used in the formation of a dental museum and library.

SEC. 8. Any person who shall practice Dentistry without having complied with the requisitions of this act, shall be deemed guilty of a violation of law, and upon conviction thereof before a magistrate, shall be fined not less than fifty nor more than two hundred dollars for each offence, which shall be collected as other debts are by law collected.

*Provided*, That nothing in this act shall be construed so as to prevent physicians and surgeons from extracting teeth.

SEC. 9. All fines imposed and collected under the provisions of this act, shall be payable one-half into the County Treasury, where such conviction shall take place, for the use of the common school fund, and one half into the Iowa State Dental Museum and Library fund.

SEC. 10. This act shall go into effect January 1st, A. D. 1871.

